# MicroRNA-18a Attenuates DNA Damage Repair through Suppressing the Expression of Ataxia Telangiectasia Mutated in Colorectal Cancer

**DOI:** 10.1371/journal.pone.0057036

**Published:** 2013-02-21

**Authors:** Chung-Wah Wu, Yu-Juan Dong, Qiao-Yi Liang, Xin-Qi He, Simon S. M. Ng, Francis K. L. Chan, Joseph J. Y. Sung, Jun Yu

**Affiliations:** 1 Institute of Digestive Disease and Department of Medicine and Therapeutics, Li Ka Shing Institute of Health Sciences, The Chinese University of Hong Kong, Hong Kong, Special Administrative Region, People’s Republic of China; 2 CUHK Shenzhen Research Institute, The Chinese University of Hong Kong, Shenzhen, People’s Republic of China; 3 Department of Surgery, The Chinese University of Hong Kong, Hong Kong, Special Administrative Region, People’s Republic of China; 4 Department of Surgery, The First Affiliated Hospital at Hebei Medical University, Shijiazhuang, People’s Republic of China; The University of Hong Kong, China

## Abstract

**Background:**

miR-18a is one of the most up-regulated miRNAs in colorectal cancers (CRC) based on miRNA profiling. In this study, we examined the functional significance of miR-18a in CRC.

**Methods:**

Expression of miR-18a was investigated in 45 CRC patients. Potential target genes of miR-18a were predicted by *in silico* search and confirmed by luciferase activity assay and Western blot. DNA damage was measured by comet assay. Gene function was measured by cell viability, colony formation and apoptosis assays.

**Results:**

The up-regulation of miR-18a was validated and confirmed in 45 primary CRC tumors compared with adjacent normal tissues *(*p*<*0.0001). Through *in silico* search, the 3′UTR of *Ataxia telangiectasia mutated (ATM)* contains a conserved miR-18a binding site. Expression of ATM was down-regulated in CRC tumors (p*<*0.0001) and inversely correlated with miR-18a expression (r = -0.4562, p*<*0.01). Over-expression of miR-18a in colon cancer cells significantly reduced the luciferase activity of the construct with wild-type ATM 3′UTR but not that with mutant ATM 3′UTR, inferring a direct interaction of miR-18a with ATM 3′UTR. This was further confirmed by the down-regulation of ATM protein by miR-18a. As ATM is a key enzyme in DNA damage repair, we evaluated the effect of miR-18a on DNA double-strand breaks. Ectopic expression of miR-18a significantly inhibited the repair of DNA damage induced by etoposide (p*<*0.001), leading to accumulation of DNA damage, increase in cell apoptosis and poor clonogenic survival.

**Conclusion:**

miR-18a attenuates cellular repair of DNA double-strand breaks by directly suppressing ATM, a key enzyme in DNA damage repair.

## Introduction

miRNAs are 18- to 25-nucleotide non-coding RNA molecules that regulate mRNA translation. They exert the effects by targeting the RNA-induced silencing complex (RISC) to complementary sites in the 3′ untranslated region (UTR) of their target genes [1]. Binding of a miRNA-loaded RISC to a complementary sequence will lead to either translational repression or decay of the targeted mRNA [2]. Through this, miRNAs regulate a variety of cellular processes including apoptosis [3], [4], differentiation [5] and cell proliferation [6]. Altered miRNA expression profiles were found in most tumor types including colorectal cancer (CRC) [7], [8], [9], [10]. Manipulation of specific miRNAs was found to be able to modulate tumor development in animal model [6], [11], [12]. Previously, through profiling the expression of 667 miRNAs in human colorectal cancer tissues, we identified miR-18a as one of the most up-regulated miRNAs in human CRC [13]. A high level of miR-18a can be detected in stool of CRC patients compared to individuals with normal colonoscopy. Upon removal of the tumor, stool level of miR-18a dropped significantly [13].

miR-18a belongs to the miR-17-92 cluster, which is located at chromosome 13q31.1 region. The oncogenic role of the miR-17-92 cluster is well documented. Over-expression of the cluster is associated with accelerated tumor growth [6] and cell proliferation [14]. Chromosomal copy number gain at the miR-17-92 cluster region was associated with the neoplastic progression from adenoma to carcinoma [15]. High expression of miR-18a has been implicated in breast cancer [16], bladder cancer [17] and pancreatic cancer [18]. However, the functional role of miR-18a in CRC remains unclear. In this study, we aimed to identify its target gene and its critical role in CRC.

## Methods and Materials

### Human Tissue Samples

Rectal tumor and adjacent non-tumorous tissues were obtained from 45 patients with histologically-confirmed rectal cancer when they underwent surgery at the Prince of Wales Hospital, Hong Kong during 1999 to 2003. Normal rectal mucosa was obtained from healthy controls during colonoscopy at the Prince of Wales Hospital during 2009. All subjects provided their written informed consent prior to specimen collection. The study protocol and consent procedure were approved by the Ethics Committee of The Chinese University of Hong Kong.

### Cell Culture, miRNA Precursors and Transfection

CRC cell lines HCT-116 and HT-29 were adopted for *in vitro* assays because these two cell lines express functional ATM in response to DNA double-strand break (DSBs) [19], [20]. Both cell lines were purchased from the American Type Culture Collection and cultured in McCoy's 5A medium (Sigma-Aldrich, St Louis, MO) with 10% fetal bovine serum (Invitrogen, Carlsbad, CA) in 5% CO_2_ at 37°C. The precursor of miR-18a (pre-miR-18a) and negative control (pre-miR-ctrl) were purchased from Applied Biosystems (Foster City, CA). Transfection was carried out using Lipofectamine 2000 (Invitrogen) according to manufacturer’s guide.

### Dual-luciferase Reporter Assay

The potential miR-18a binding site in ATM 3′ untranslation region (3′UTR) was predicted by targetscan (www.targetscan.org) and miRanda (www.microRNA.org). Sequences with the wild-type or mutant seed regions were cloned into pMIR-REPORT luciferase vector (Applied Biosystems). The mutant ATM 3′UTR sequence was prepared by mutating 5 nucleotides in the seed region. The synthesized oligos were shown as follows:

Wild-type sense strand:

5′-CTAGTTGTGTCCCAATTTCAAGTATTTTAATTGCACCTTAATGAAATTATCGAGCT-3′.

Wild-type anti-sense strand:

5′-CGATAATTTCATTAAGGTGCAATTAAAATACTTGAAATTGGGACACAA-3′.

Mutant sense strand:

5′-CTAGTTGTGTCCCAATTTCAAGTATTTTAATTTCGTATCAATGAAATTATCGAGCT-3′.

Mutant anti-sense strand:

5′-CGATAATTTCATTGATACGAAATTAAAATACTTGAAATTGGGACACAA-3′.

The cell lines transiently transfected with pre-miR-18a or pre-miR negative control (at 15 nM final concentration) in 24-well plates were co-transfected with *Renilla* luciferase report vector (195 ng/well) and *Firefly* luciferase vector (5 ng/well) using Lipofectamine 2000 (Invitrogen). Cells were harvested 48 hours posttransfection and luciferase activities were analyzed by the dual-luciferase reporter assay system (Promega, Madison, WI).

### miRNA Quantitation by Quantitative Reverse Transcription Polymerase Chain Reaction

Quantitative reverse transcription polymerase chain reaction (qRT-PCR) of individual miRNA was performed using the TaqMan miRNA reverse transcription kit (Applied Biosystems) and the TaqMan human miRNA assay (RNU6B: 001093; miR-18a: 002422; miR-16: 000391) based on a modified protocol from Applied Biosystems [21]. miRNA expression level was normalized to internal control. The experiment operators were unaware of the clinical data at the time the quantitation of miRNA was carried out.

### qRT-PCR for mRNA

For ATM mRNA quantitation, total RNA was reverse transcribed with random primer using Reverse Transcription Kit (Applied Biosystems), and real-time PCR was set with Power SYBR Green PCR Master Mix (Applied Biosystems). ATM expression was normalized to glyceraldehyde-3-phosphate dehydrogenase (GAPDH) Primer sequences are as follows: ATM: Forward: 5′- GGAGAGCTGGAAAGCATTGG-3′; Reverse: 5′- TGAGAAGCTGGGAGTGTTTCTG-3′. GAPDH: Forward: 5′-GAAGGTGAAGGTCGGAGT-3′ Reverse: 5′-GAAGATGGTGATGGGATTTC-3′.

### Western Blot Analysis

Total protein was extracted and protein concentration was measured by the Bradford DC protein assay (Bio-Rad, Hercules, CA). 20 to 40 µl of protein from each sample were separated on 8% Bis/Tris-polyacrylamide gel through electrophoresis and blotted onto nitrocellulose membranes (GE Healthcare, Piscataway, NJ). Blots were immunostained with primary antibodies at 4°C overnight and secondary antibody at room temperature for 1 hour. Anti-ATM (2C1) antibody was purchased from Genetex (Irvine, CA). Anti-phospho-Checkpoint kinase 2 antibody (Thr68) was purchased from Cell Signaling (Danvers, MA). Anti-GAPDH (SC-25778) antibody was purchased from Santa Cruz Biotechnology (Santa Cruz, CA).

### Comet Assay

HCT-116 cells were transiently transfected with pre-miR-18a or pre-miR negative control (at 15 nM final concentration) in 24-well plates. After one hour of incubation with 2 µM etoposide or DMSO, the cells were either harvested or switched to medium without etoposide for 2 hours to allow DNA damage repair. At the end of the experiment, cells were harvested for comet assay according to the manufacturer’s guide (Trevigen, Gaithersburg, MD). The tail moments of the cell comets were analysed automatically using a comet analysis software, CometScore (Tri Tek Corp, Sumerduck, VA). Tail moments of 50 or more cells per slide were determined.

### Colony Formation and Cell Viability Assay

Cells (1×10^5^ per well) were plated in a 24-well plate and transfected with pre-miR-18a or pre-miR-ctrl at 15 nM. Twenty-four hours after transfection, cells were incubated with 2 µM etoposide or DMSO for 1 hour, collected and seeded (500–1000/well) in a fresh 24-well plate for 9 days. Colonies were counted after staining with *Harris hematoxylin* solution. Cell viability was determined by the 3-(4,5-Dimethylthiazol-2-yl)-2, 5-diphenyltetrazolium bromide (MTT) assay (Promega) according to manufacturer’s guide. Briefly, MTT solution was added to each well at a final concentration of 1 mg/ml per well and the plates were incubated at 37°C for another 3 h. After incubation, 200 µl of DMSO was added to each well to dissolve the formazan formed and the absorbance was read at 570 nm using a spectrophotometer. All experiments were triplicated.

### Annexin V Apoptosis Assay

Cells (1×10^5^ per well) were seeded in a 24-well plate and transfected with pre-miR-18a or pre-miR-ctrl at 15 nM. Twenty-four hours after transfection, cells were incubated with 2 µM etoposide or DMSO for 1 hour. Apoptosis was assessed by flow cytometry after staining with Annexin V (FITC-conjugated) (BD Biosciences, Erembodegem, Belgium) and 7-amino-actinomycin (7-AAD; BD Biosciences).

### Statistics

Association between miR-18a and ATM expression was analyzed by Spearman r correlation test. Difference between two groups in luciferase reporter assay, comet assay and colony formation assay was determined by student t-test. Associations of miR-18a expression level with clinicopathological features were analyzed by Fisher exact test. Regression analysis was analyzed by SPSS (IBM, New York, US). Difference in cell growth curves was determined by repeated measures ANOVA. Progression-free survival analysis was done by the Kaplan–Meier method and the results were tested using the Log-rank test. p<0.05 was taken as statistical significance. All statistical tests except regression analysis were done by Graphpad Prism 5.0 (Graphpad Software Inc., San Diego, CA).

## Results

### miR-18a is Up-regulated in Rectal Tumor

**Figure 1:**
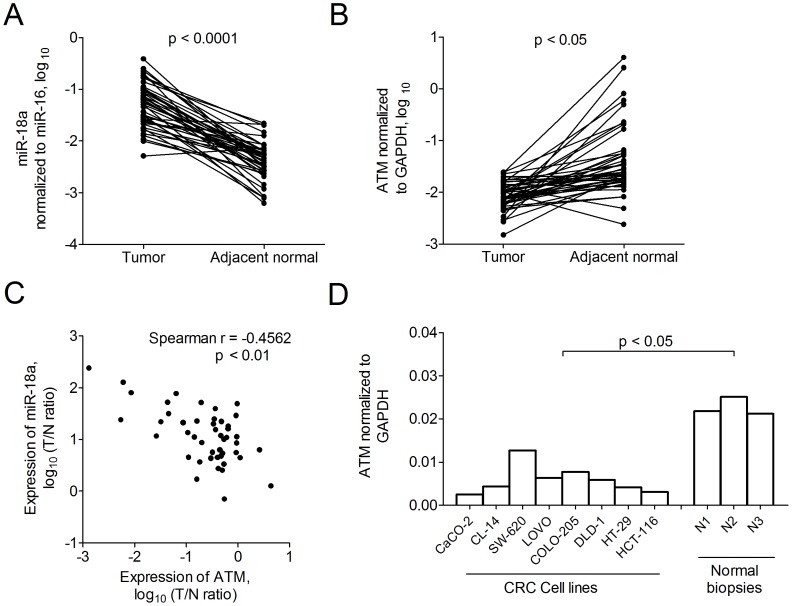
Expression of miR-18a in colorectal cancer. Expression of (A) miR-18a, normalized to miR-16a and (B) ATM, normalized to GAPDH in 45 pairs of rectal tumors and adjacent normal tissues. p values indicate significant differences between paired samples determined by the Wilcoxon matched pairs test. (C) Scatter plots showing the association between miR-18a level and ATM expression. (D) Expression of ATM normalized to GAPDH in 8 colorectal cell lines and three normal colon biopsies (N1, N2 and N3).

**Figure 2:**
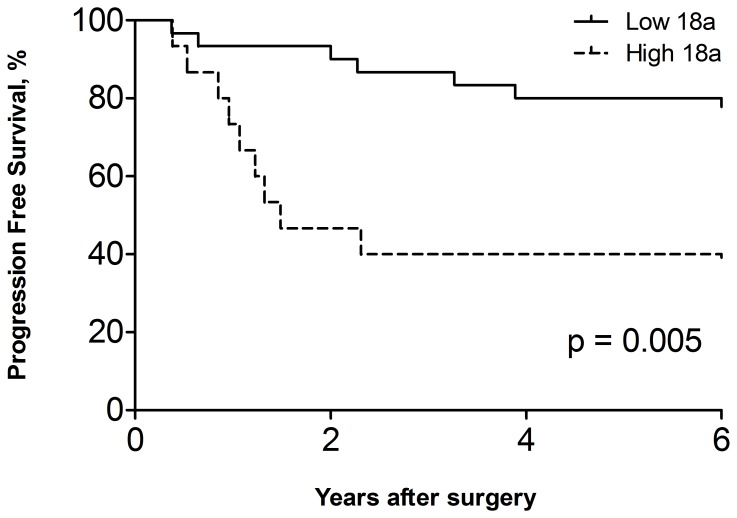
Progression-free survival of rectal cancer patients stratified based on tumor miR-18a expression. High expression of miR-18a in tumors was defined based on the highest tertile. miR-18a expression was normalized to that of miR-16 and referenced to miR-18a level in adjacent normal tissue. p value was determined by Log-rank test.

Among the 45 pairs of rectal cancer tissue samples, 44 pairs had higher miR-18a expression in tumor than in adjacent normal tissue (p<0.0001; Figure 1A), with a median difference of 11.46-fold increase (IQR 4.73–26.00). A high expression of miR-18a in tumor was associated with higher recurrence rate, of which 9 out of 15 cases with high tumor miR-18a recurred compared to only 6 out of 29 cases with low tumor miR-18a recurred after surgical resection (p<0.05, table 1). Multivariate analysis further confirmed that the association between miR-18a level and recurrence was independent to age, gender and cancer stage (Table 1). Progression-free survival analysis showed that patients with high tumor miR-18a level tend to have quicker recurrence after surgery, compared to patients with low tumor miR-18a level (p* = *0.005, Figure 2).

**Table 1:** Association of miR-18a expression with clinicopathological features of rectal cancer patients.

	miR-18a expression in tumors^a^		p values	
	low	high	Univariate analysis^b^	Multivariate analysis^c^
Age at enrollment, cases			1.000	0.329
<65 (n = 18)	12	6		
≥65 (n = 27)	18	9		
Gender, cases			0.110	0.082
Women (n = 17)	14	3		
Men (n = 28)	16	12		
Duke stage, cases			1.000	1.000
A and B (n = 26)	17	9		
C (n = 19)	13	6		
Recurrence, cases			0.023	0.015
No (n = 29)	23	6		
Yes (n = 16)	7	9		

a High expression of miR-18a in tumors was defined based on the highest tertile. miR-18a expression was normalized to that of miR-16 and referenced to miR-18a level in adjacent normal tissue.

b Univariate analysis was analyzed by Fisher exact test.

c Multivariate analysis was analyzed by binary logistic regression that enter all clinical covariates in a single step.

### In Silico Prediction of miR-18a Target and Validation by Luciferase Assay

**Figure 3:**
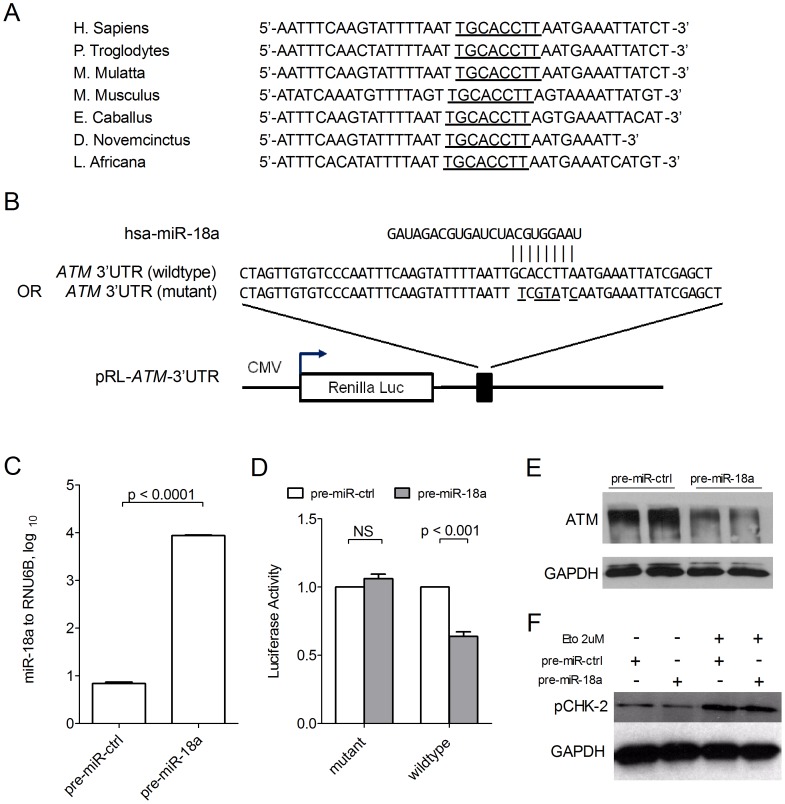
Regulation of miR-18a on the 3′-UTR of ATM. (A) The miR-18a alignment site (underlined) within ATM 3′UTR of different species is conserved. (B) Mature human miR-18a sequence and region of 3′-UTR of human ATM containing the recognition site, which was cloned into a construct with *Renilla* luciferase. The mutant 3′-UTR contains a seed region with 5 mutated nucleotides (underlined). (C) Expression of miR-18a in HCT-116 cells transfected with miRNA precursor control (pre-miR-ctrl) or miR-18a precursor (pre-miR-18a) at 15 nM. (D) Luciferase activity in HCT-116 cells co-transfected with the *Renilla* luciferase construct (containing wildtype or mutant miR-18a seed region), *Firefly* luciferase construct and pre-miR-ctrl or pre-miR-18a at 15 nM. Level of activity was calculated by normalizing *Renilla* luciferase to *Firefly* luciferase. NS denotes no statistical significance. p value was determined by student t-test. Mean and standard deviation (SD) was calculated from three independent experiments. (E) Immunoblot of endogenous ATM expression in HCT-116 cells 48 hours after transfection of pre-miR-ctrl and pre-miR-18a. (F) Immunoblot of the phosphorylated form of CHK-2 protein, a direct downstream target of ATM, in HCT-116 cells, after transfection of pre-miR-ctrl or pre-miR-18a under the treatment with DMSO or 2 µM etoposide for 1 hour.

Using algorithms for target gene prediction, TargetScan [22] and miRanda [23], the key enzyme in DNA damage repair, Ataxia Telangiectasia Mutated (ATM), was identified as one of the potential targets of miR-18a. The sequence alignment of miR-18a with different species of ATM 3′UTR was also conserved (Figure 3A), indicating that ATM is one of the potential direct targets of miR-18a. The predicted binding of miR-18a with *Homo sapiens* ATM 3′UTR is illustrated in Figure 3B. To further confirm that ATM is the direct target of miR-18a, a segment of the 3′UTR of ATM consisting the seed region, with or without point mutations, was sub-cloned downstream of the firefly luciferase reporter (Figure 3B). The constructs were then co-transfected with pre-miR-18a or with pre-miR control for luciferase activity assays. Ectopic expression of pre-miR-18a in HCT-116 cells was confirmed by qRT-PCR (p<0.0001, Figure 3C). The relative luciferase activity of the wild-type construct of ATM 3′UTR in HCT-116 was significantly reduced in the presence of miR-18a (p<0.001; Mann-Whitney test), whereas such a suppressive effect of miR-18a on luciferase activity was not observed in the presence of mutant ATM 3′UTR (Figure 3D), indicating a direct and specific interaction of miR-18a on ATM 3′UTR. The interaction was further confirmed by the observation that over-expression of miR-18a was able to reduce ATM protein level and the phosphorylation of Checkpoint Kinase (CHK-2), a direct downstream phosphorylation target of ATM *in vitro* (Figure 3E). Under etoposide treatment for 1 hour, pCHK-2 level was significantly up-regulated regardless of miR-18a over-expression.

### ATM is Down-regulated in Rectal Tumor and CRC Cell Lines

ATM expression was evaluated in 45 pairs of rectal tumor and adjacent normal tissues. In contrast to miR-18a, expression of ATM was significantly lower in tumors than in non-tumor tissues (p<0.0001; Figure 1B), with a median difference of 0.369-fold (IQR 0.127–0.575). Expression of miR-18a and that of ATM were inversely correlated with a spearman r = -0.4562 (p<0.01; Figure 1C). The aberrant down-regulation of ATM was also observed in CRC cell lines compared with the normal colon biopsies (p<0.05, Figure 1D).

### miR-18a Regulates Double-strand DNA Damage Recovery

**Figure 4:**
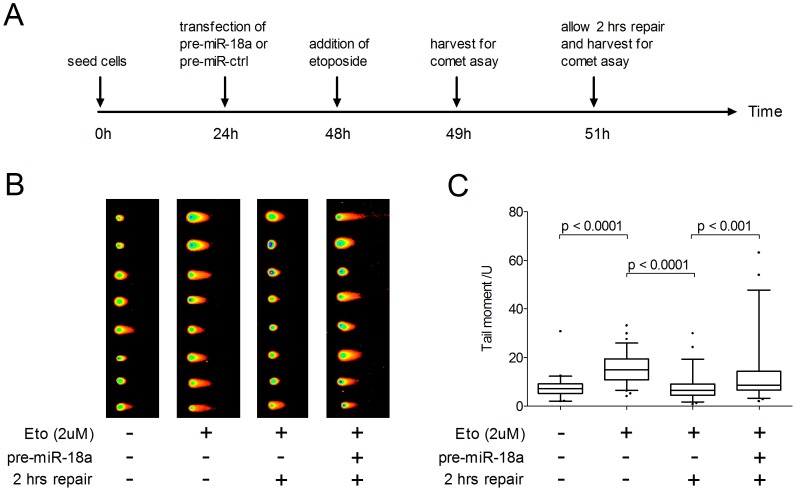
Measurement of DNA damage in HCT-116 cells using comet assay. (A) The timeline of the order of treatment (B) Samples of comet tails from each group, from left to right, cells transfected with miRNA control precursor (pre-miR-ctrl) and without drug treatment, cells transfected with pre-miR-ctrl and treated with 2 µM etoposide, cells transfected with pre-miR-ctrl and treated with 2 µM etoposide, and cells transfected with pre-miR-18a and treated with 2 µM etoposide, last two groups of cells were allowed for two hours in normal medium after drug treatment. (C) Box plot of tail moment of cells under different treatment based on analyzing 50 cells from random microscopic fields. The box represents the interquartile range, the line across the box indicates the median values and whiskers represent 5–95 percentile values. p values were evaluated by non-parametric t-test.

ATM activation represents an early and important event for DNA repair in reponse to DNA DSBs [24]. As miR-18a is able to suppress ATM expression, we hypothesize the over-expression of miR-18a in cells would inhibit the recovery mechanism. The level of DSBs was investigated by comet assay. Without the induction of DNA damage, HCT-116 cells had a baseline tail moment of 7.75±4.37 units. Exposure to 2 µM etoposide for 1 hour resulted in a significantly higher tail moment (15.46±6.07 units, p<0.0001), indicating the induction of DNA DSBs by etoposide. When allowed for 2 hours in normal medium supplemented with 10% FBS for recovery, tail moment of etoposide treated HCT-116 cells restored to baseline level (7.69±5.14 units), whereas tail moment of HCT116 cells over-expressing miR-18a remained significantly higher than baseline level (p<0.001), indicating miR-18a induced an effect of prohibiting DNA repair (Figure 4).

### miR-18a Increases Sensitization of CRC Cells to Genotoxin

**Figure 5:**
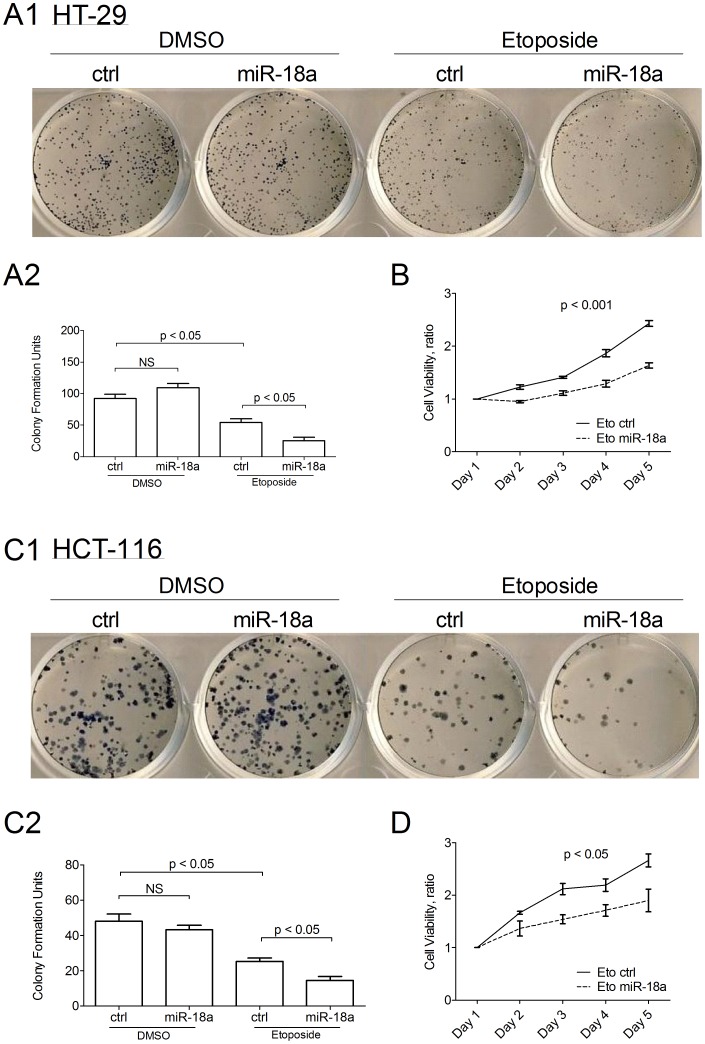
Effect of ectopic miR-18a expression on cellular sensitivity to genotoxic agent. The effect of ectopic expression of miR-18a on cell growth of (A) HT-29 cells or (C) HCT-116 cells, with or without treatment of 2 µM etoposide. Mean±SD was calculated from three independent experiments. Significant difference was determined by student t-test. NS denotes no statistical significance. Effect of miR-18a on (B) HT-29 and (D) HCT-116 cell viability after treatment with 2 µM etoposide. Mean±SD was calculated from three independent experiments. Significant difference was determined by repeated measure ANOVA.

Without prompt response and repair, DNA damage accumulates and reduces cell growth and cell viability. We investigated the effect of miR-18a on CRC cell growth by colony formation assay in two CRC cell lines (HT-29 and HCT116). Without the induction of DNA damage, over-expression of miR-18a did not have significant effect in cell growth as compared to cells transfected with precursor control both in HT-29 (Figure 5A) and in HCT-116 (Figure 5C). Exposed to 2 µM etoposide, the formed colonies were significantly reduced in HT-29 (Figure 5A) and in HCT-116 (Figure 5C). Consistently, ectopic expression of miR-18a significantly suppressed cell viability as compared to precursor control over-expressing HT-29 (p<0.001; Figure 5B) and HCT-116 (p<0.05; Figure 5D).

### miR-18a Promotes Genotoxin Induced Apoptosis

**Figure 6:**
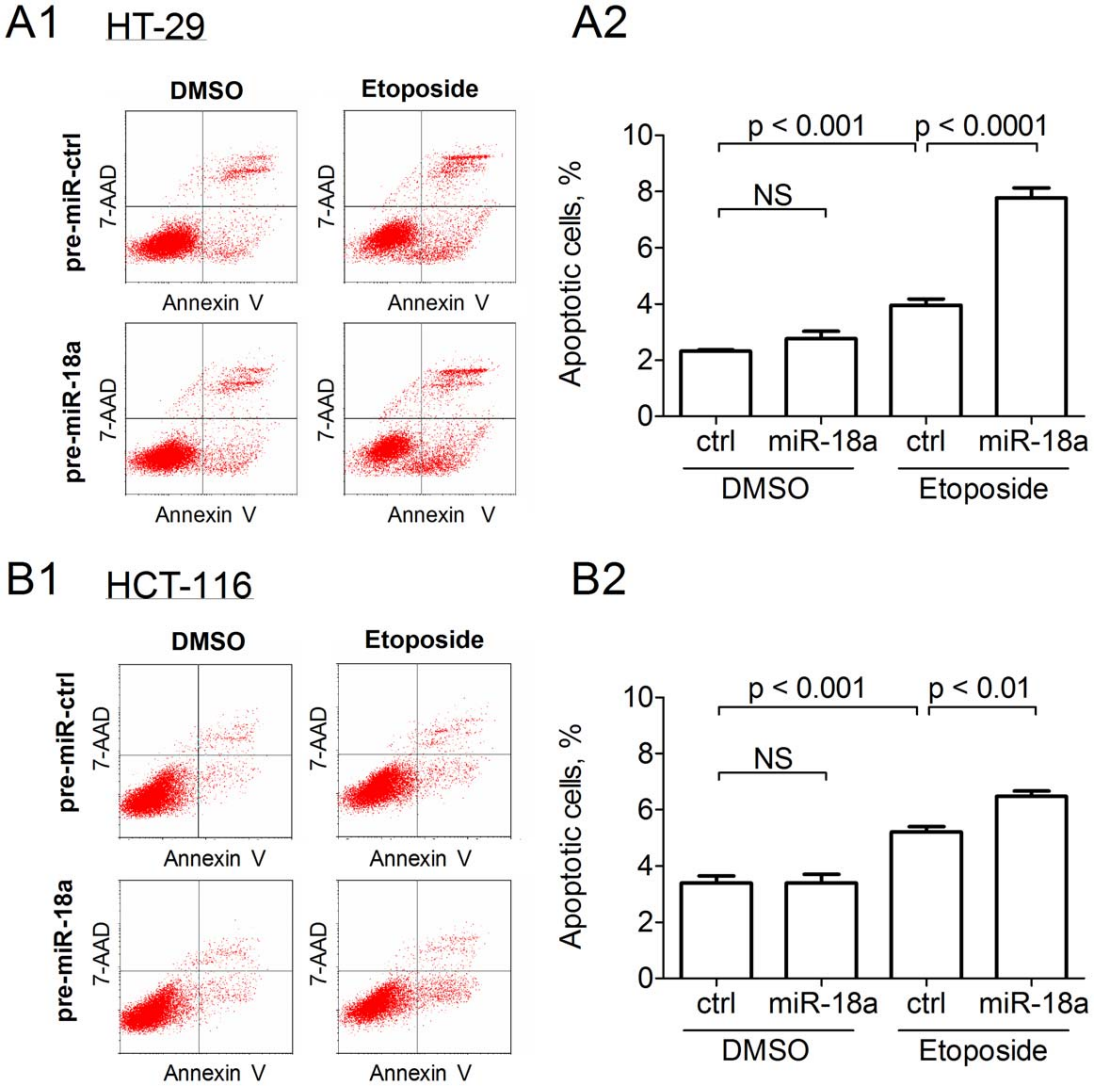
The effect of ectopic miR-18a expression on apoptosis. (A) HT-29 cells or (B) HCT-116 cells, with or without treatment of 2 µM etoposide. Mean±SD was calculated from three independent experiments. Significant difference was determined by student t-test. NS denotes no statistical significance.

Without the induction of DNA damage, over-expression of miR-18a did not induce significant effect in cell apoptosis in HT-29 and in HCT-116 (Figure 6). Exposure to 2 µM etoposide significantly induced the amount of apoptotic cells in both HT-29 and HCT-116 cells (both p<0.001; Figure 6A2 and 6B2). Over-expression of miR-18a further induced apoptosis synergistically with etoposide in both HT-29 (p<0.0001) and HCT-116 (p<0.01) cells.

## Discussion

In this study, a clear connection between miR-18a and the gene ATM was established. Luciferase reporter assay and western blot analysis confirmed the interaction, which was through the binding of miR-18a to the 3′ untranslated region of ATM mRNA and subsequently suppressed its protein translation and its activity. This association was most evident from the inverse correlation between miR-18a and ATM in rectum tumor tissues (p<0.01).

ATM is a high molecular weight protein kinase that plays a central and early role in promoting repair of DNA DSBs, which are one form of the most cytotoxic DNA lesions that arise through both endogenous (e.g. oxidative stress) and exogenous (e.g. ionizing radiation and genotoxic agents) sources. In unstimulated cell, ATM mostly exists as an inactive homodimer or multimer, with the kinase domain of one ATM protein bound to the internal domain of another ATM protein containing the serine 1981-phosphorylation site [24]. This structure is essential to keep ATM protein inactive and stable when there is no DNA damage. Therefore, under no external stimulus that leads to DNA damage, miR-18a over-expression induced no significant phenotypic change in HT-29 and HCT-116 cells as evident by cell viability, proliferation and apoptosis analysis compared with control groups. However, in response to DNA damage induced by etoposide, a genotoxic agent that specifically induces DSBs, we found that cells over-expressing miR-18a were less able to restore from damage compared to cells transfected with miRNA control precursor, reflecting a compromised DNA DSBs repair mechanism. Under DNA damage stimulus, the kinase domain of one ATM protein phosphorylated the 1981-domain of the interacting ATM protein, resulting in active kinase in monomeric form [24]. Activated ATM is freed and can phosphorylate a diverse array of downstream targets that participate in events to repair the DNA damage [25]. Over-expression of miR-18a reduced ATM protein amounts and thus the availability of activated ATM for DNA repair. Therefore, as DNA damage accumulates without prompt repair, miR-18a over-expressing cells were more prone to commit apoptosis, reduced clonogenic survival and proliferation rate, as evident in both HT-29 and HCT-116 cells.

Compromised DNA repair mechanism due to loss of ATM function is a known predisposition to various diseases. The most evident example being the inherited autosomal recessive disorder, Ataxia-telangiectasia (A–T), which results from loss of ATM protein expression or functional protein product. The disease is characterized by progressive cerebellar ataxia, neuro-degeneration, radiosensitivity, cell-cycle checkpoint defects, genome instability, and a predisposition to various forms of cancer [26], [27], [28]. Chromosomal gain in region 13q31.1, where miR-17-92 is located, is an early event in the adenoma-carcinoma sequence. Consistently, up-regulation of miR-18a is found since precancerous stage of CRC [13]. The suppressed DNA repair mechanism induced by up-regulated miR-18a could possibly serve a catalyzing role in the formation of carcinoma.

Currently, tumor stage is the most important prognostic indicator for CRC patients. Nevertheless, many patients developed recurrence after surgical resection regardless of stage or the provision of adjuvant chemotherapy. Additional prognostic biomarkers are needed to provide better recurrence risk assessment so patients can benefit from close follow-up. We found high miR-18a level is associated with higher recurrence rate and quicker recurrence. It remains to be elucidated whether this phenomenon is mediated through ATM or other miR-18a target genes. Nevertheless, the role of ATM in predicting chemo-/radio-therapy resistance in a clinical setting remains not clearly established. Roossink et al. reported that ATM activation induced protective role to chemo−/radio-therapy in a cohort of cervical cancer patients [29]. Jiang et al., however, showed that ATM could sensitize and protect against doxorubicin-induced cytotoxicity, depending on the proficiency of other DNA repair genes such as p53 and CHK-2 [30]. Huehls et al. showed that depletion of ATM did not sensitize cells to 5-FU, which is the main regimen used in CRC [31]. Admansen et al. also showed that at clinical relevant dosage of 5-FU, the ATM-pathway is not activated for DNA repair in CRC cells [32]. Therefore, though our *in vitro* data clearly demonstrated the suppression of ATM by miR-18a sensitized cancer cells to etoposide, the role of ATM role on chemo-resistance may vary in a chemotherapeutic-specific and tumor-specific manner *in vivo*. Besides, miR-18a-associated recurrence can also be mediated through other potential target genes that induce its oncogenic nature *in vivo*. This hypothesis, however, needs further investigation and validation. The establishment of miR-18a as recurrence marker also needs to be validated in a cohort of larger sample size.

In conclusion, we identified ATM, a protein crucial to DNA repair, as the target of miR-18a. In rectal cancer tissues, expression of miR-18a and ATM correlated inversely. Ectopic expression miR-18a suppresses ATM expression and attenuates DNA DSB repair. miR-18a, a frequently up-regulated miRNA in CRC, induces its oncogenic effect at least partly through suppressing ATM. Moreover, tumor miR-18a level is a potential marker for rectal cancer recurrence.
